# Suppression of Berberine and Probiotics (*in vitro* and *in vivo*) on the Growth of Colon Cancer With Modulation of Gut Microbiota and Butyrate Production

**DOI:** 10.3389/fmicb.2022.869931

**Published:** 2022-04-28

**Authors:** Chao Huang, Ying Sun, Sheng-rong Liao, Zhao-xin Chen, Han-feng Lin, Wei-zeng Shen

**Affiliations:** Department of Traditional Chinese Medicine, The Second Affiliated Hospital of Shenzhen University, People’s Hospital of Shenzhen Baoan District, Shenzhen, China

**Keywords:** colon cancer, gut microbiota, berberine, probiotics, butyrate

## Abstract

**Background and Objective:**

An increasing number of evidence has revealed that the gut microbiome functions in immunity, inflammation, metabolism, and homeostasis and is considered to be crucial due to its balance between human health and diseases such as cancer, leading to the emergence of treatments that target intestinal microbiota. Probiotics are one of them. However, many challenges remain regarding the effects of probiotics in cancer treatment. Berberine (BBR), a natural extract of Rhizoma Coptidis and extensively used in the treatment of gastrointestinal diseases, has been found to have antitumor effects *in vivo* and *in vitro* by many recent studies, but its definite mechanisms are still unclear. This study aimed to explore the inhibitory effect of BBR and probiotics on the growth of colon cancer cells *in vitro* and *in vivo*, and the regulatory influence on the gut microbiome and butyrate production.

**Methods:**

Colon cancer cell line HT29 was used to establish a xenograft model of nude mice and an *in vitro* model. A total of 44 nude mice and HT29 cells were divided into control, model, model + BBR, model + probiotics, and model + combination of BBR with probiotics (CBPs). Live combined *Bifidobacterium*, *Lactobacillus*, and *Enterococcus* powder (LCBLEP) was used as a probiotic preparation. LCBLEP was cultured in the liquid medium under anaerobic conditions (the number of viable bacteria should reach 1 × 10^8^CFU), and the supernatant was collected, and it is called probiotic supernatant (PS). Model + BBR and model + probiotics groups were treated with BBR and LCBLEP or PS for 4 weeks *in vivo* or 48, 72, and 96 h *in vitro*, respectively. Tumor volume or cell proliferation was measured. Gut microbiota was pyrosequenced using a 16S rDNA amplicon. HDAC1 mRNA level in HT29 cells and sodium butyrate (SB) expression in the serum of mice was detected by QPCR and ELISA.

**Results:**

The treatment of BBR and CBP reduced the growth of neoplasms in mice to a different extent (*p* > 0.05), especially at 14 days. The inhibitory effect of LCBLEP on tumor growth was more significant, especially at 11–21 days (*p* < 0.05). Inhibition of BBR on *in vitro* proliferation was concentration-dependent. The suppression of 75% probiotic supernatant (PS) on the proliferation was the most significant. The supplement of LCBLEP significantly increased the richness and evenness of the gut microbe. BBR dramatically increased the abundance of *Bacteroidete*s and *Proteobacteria*, with reduced *Ruminococcus*, followed by the LCBLEP. The LCBLEP reduced the relative abundance of *Verrucomicrobia* and *Akkermansia*, and the CBP also promoted the relative level of *Bacteroidete*s but reduced the level of *Verrucomicrobia* and *Akkermansia*. BBR and LCBLEP or CBP improved the alpha and beta diversity and significantly affected the biomarker and metabolic function of the gut microbe in nude mice with colon cancer. The level of HDAC1 mRNA was reduced in HT29 cells treated with BBR or PS (*p* < 0.05), the mice treated with BBR revealed a significantly increased concentration of SB in serum (*p* < 0.05), and the inhibitory effect of SB on the proliferation of HT29 cells was stronger than panobinostat and TSA.

**Conclusion:**

Although the combination of BBR and probiotics has no advantage in inhibiting tumor growth compared with the drug alone, BBR can be used as a regulator of the intestinal microbiome similar to the probiotics by mediating the production of SB during reducing the growth of colon cancer.

## Introduction

So far, cancer remains a major global killer. Colorectal cancer (CRC) is the third most common cancer type (10.2%) and the second most fatal cancer worldwide (9.2%) (Bray et al., 2018). The exploration of cancer pathogenesis and related drugs has always been the focus of research. Recently, more and more attention has been paid to the development of tumor-related microenvironments and natural drugs.

The human microbiota consists of 10–100 trillion microbes including bacteria, viruses, protozoa, and fungi, and most harbor in the gut with biomass of 1.5 kg (amount to 10^11^–10^12^cells). An increasing number of potent evidence has revealed that a strong correlation existed between the intestinal microbiome including specific microbes and the occurrence of colon cancer. Dysbacteriosis in the gut including changes in microbiome structure and function has been found to promote carcinogenesis (Fong et al., 2020; Song M. et al., 2020). In that case, reversion of the microecological imbalance becomes a novel strategy for the prevention and treatment of colon cancer. The application of probiotics is one of the many promising strategies.

In fact, the ancients first used human feces to treat some infections or food poisoning in China, which was the first description of an initiative modification of the intestine microbiota. Probiotics were first defined by Lilly and Stillwell (1965), but its restriction was only some substances produced by bacteria. The exploration never ends. Recently, probiotics were defined as living microorganisms that must be taken in sufficient quantities with a protective effect on the body’s health, not limited to nutrition (Hill et al., 2014). In this case, probiotics mainly exert biological effects through multiple functions including the influence of the resident microbiota, regulation of barrier of the gut epithelium, and mediation of the global immune. Until now, probiotics developed are grouped into many categories including *Lactobacillus*, *Bifidobacterium*, *Gram-positive cocci* (*Streptococcus faecalis*, *Lactococcus lactis*, *Streptococcus intermedius*), and *next-generation probiotics* (Satokari, 2019).

The microbes in the gut are not static, but in a steady state of dynamic change, their modifications are also regulated by multiple factors, such as inheritance, nutrition, and internal and external environments (Gomaa, 2020). The alterations in the composition and function of the gut microbiome have an influence on the intestinal barrier, digestion and metabolism, and immune responses. An increasing number of evidence has found that the disorder of gut microbiota is responsible for many types of diseases. Therefore, the improvement in gut microbiota contributed by probiotics has generated considerable interest. Because probiotics are derived from different foods or drugs, understanding probiotics would promote the development of food and pharmaceutical industries, such as commercial yogurt and prebiotic drinks.

Live combined *Bifidobacterium*, *Lactobacillus*, and *Enterococcus* powder (LCBLEP) is a commercial probiotic preparation (the brand name is Peifeikang) and is used widely in a clinic. Several research studies have demonstrated that these probiotics have the potential to reduce the enteropathogenic complications in patients with colon cancer undergoing surgery or inhibit the growth of the cancer cells through the creation of the integrity of intestinal mucosal, promotion of the immune, and production of antitumor metabolites, then preventing the progress of the CRC (Eslami et al., 2019; Bazireh et al., 2020; Sugimura et al., 2021).

Berberine (BBR), a natural plant alkaloid extracted from *Coptis chinensis* (Huanglian), has long been used to treat digestive diseases as an ancient antidiarrheal medication in China (Zhang Y. et al., 2020). In addition, BBR is also employed to treat metabolic and tumorous disorders, such as type 2 diabetes and malignancy (Zhang et al., 2014; Gong et al., 2020). Animal studies have shown that BBR significantly altered the microbiome of the intestine and microbe-related mechanisms through 16S rRNA gene sequencing (Kumar et al., 2015; Yang et al., 2017). Given the efficacy of BBR, it is considered a bacteriostatic agent and is used to treat the kinds of diseases with probiotics (Zhang Y. et al., 2020). However, it is unclear about the effect of the combination of BBR and probiotics on the growth of colon cancer and whether this efficacy is associated with the changes in the gut microbiome.

## Materials and Methods

### Animals and Reagent

Human colonic cancer cell line HT29 (JNO-21409) was obtained from Guangzhou Genio Biological Technology Co., Ltd. BALB/c nude mice aged 4–5 weeks were collected from Guangdong Yaokang Biotechnology Co., Ltd. Fetal bovine serum was purchased from Gibco (10099-141). BBR was purchased from Northeast Pharmaceutical Group Shenyang First Pharmaceutical Co. Ltd. Live combined *Bifidobacterium* (BNCC232112), *Lactobacillus* (BNCC336974) and *Enterococcus* (BNCC192631) powder (LCBLEP), MRS medium dry powder (0016), and Intestinal Bacterial Enrichment Broth (EE broth, 10206) were purchased from Guangzhou Leisha Biological Technology Co., Ltd. Nucleic acid extraction or purification reagent, Agencourt AMPure XP60ml Kit, Qubit dsDNAHS Assay Kit, NovaSeq 6000 S4 Reagent Kit (300 cycle) were purchased from Guhe of China (GHFDE100), Beckman Coulter (A63881), Life tech (Q32851), and Illumina (20012866), respectively. Mouse sodium butyrate (SB) ELISA Kit was from MEIMIAN Biotechnology Co., Ltd (MM-46099M1). A total number of three HDAC inhibitors, such as panobinostat (LBH589), sodium butyrate, trichostatin A (TSA), were obtained from Beyotime (Wuhan, China).

### Establishment of a Xenograft Model of Colon Cancer in Nude Mice

Animal feeding and management were carried out in accordance with “laboratory animal environment and facilities” (GB 14925-2010). The colon cancer cell line HT29 was conventionally cultured, and the cells were collected after digestion with trypsin until 80–90% of the culture flask was filled and resuspended with PBS to 2–5 × 10^6^/ml. The cell suspension with 0.2 ml was inoculated subcutaneously on the back of nude mice for 4 weeks. Tumor growth was observed weekly and tumor size was also measured. All animals’ performances were conducted in accordance with animal ethics.

### Treatment of Berberine and Probiotics *in vitro* and *in vivo*

#### In vitro

After the mixed probiotics (Bifidobacterium, Lactobacillus acidophilus, and Enterococcus faecalis) were cultured in the liquid medium under anaerobic conditions (the number of viable bacteria should reach 1 × 10^8^CFU), the culture supernatant was collected, and it is probiotic supernatant (PS). HT29 cells were treated with 120 μmol/L, 280 μmol/L, 420 μmol/L BBR, 22.1 μmol/L cetuximab, and 25, 50, and 75% PS (diluted with culture medium) for 48, 72, 96 h, respectively. Morphological changes were observed using an inverted phase-contrast microscope (BX53M, Olympus) (10 × 20). The cell proliferation rate of each group was detected by methylthiazolyldiphenyl-tetrazolium bromide (MTT).

#### In vivo

A number of 44 BALB/c nude mice (half male and half female) were randomly divided into five groups, namely, normal control (NC group, *n* = 8), model group (*n* = 9), model + BBR (*n* = 9), model + LCBLEP (Model + LCBLEP, *n* = 9), and model + Combination of BBR with LCBLEP (Model + CBP, *n* = 9). In the model group, the xenograft model was constructed according to the above method. In the Model + BBR and Model + LCBLEP groups, the mice were, respectively, treated with BBR (78 mg/kg) and LCBLEP (7.8 × 10^6^ CFU/kg) by gavage at the beginning of modeling. A normal control group was injected subcutaneously with the same volume of normal saline. At the end of modeling, the feces of mice were collected for gut microbiome detection. Then, the nude mice were anesthetized by intraperitoneal injection of chloral hydrate to obtain tumor tissues and then sacrificed through cervical dislocation. There was no abnormal death of animals in the process of modeling.

### Detection of Cell Proliferation by CCK-8 or Methylthiazolyldiphenyl-Tetrazolium Bromide (MTT)

After the cells that were plated into 96-well plates were cultured, MTT solution was added to each well (final concentration 0.5 mg/ml) and incubated at 37°C for 4 h. The culture supernatant was removed from the well, and 150 μl DMSO was added. OD values were detected at 490-nm wavelength. Alternatively, the wells were added with 100 μl of CCK-8 solution (CK04-500, Dojindo, Japan) to each well and cultured at 37°C for 2 h, and then, OD values were detected at 450-nm wavelength.

### ELISA Assay of Sodium Butyrate in Mice Serum

The mice were killed through disconnecting cervical vertebra and the corresponding serum samples were obtained. In total, 10 μl sample with 40 μl sample diluent were added into each well of the enzyme plate which has been precoated with mice SB-specific monoclonal capture antibody. Then, 100 μl horseradish peroxidase (HRP)-labeled mice SB antibody was added and incubated at 37°C for 1 h, and the plate was washed 3 times. Color developer solution was added at 100 μl/well and incubated at room temperature for 15 min. After 50 μl/well stop buffer was added for 5 min, OD values were detected at 450-nm wavelength.

### DNA Extraction

Feces from nude mice were collected and stored in a special sample preservation solution. Nucleic acid extraction reagent (Guhe Biological Co., Ltd., Hangzhou, China, GHFDE100) was used for DNA extraction, and the NanoDrop luminance meter (Thermo Fisher Scientific, Waltham, MA, United States) was used to determine the concentration and quality of DNA.

### Sample Amplification and Electrophoresis

Nuclease-free water was used to dilute the primers to 1 μm and gDNA to 5 ng/μl. The primers (F: Illumina_uni_sequence-Read1_sequnce_GTGCCAGCMGCCGCGGTAA, R: llumina_ uni_sequence_(barcode)_read2_sequence_GGACTACHVGGGT WTCTAAT) used for PCR had been fused with the V4 universal primers of Illumina sequencing platform. A total of 50 μl PCR reaction system was prepared using Phusion High-Fidelity PCR Master Mix with HF buffer, and PCR amplification was performed. Then, PCR products were detected by 1% agarose gel electrophoresis with a sample loading of 2 μl.

### Purification of Magnetic Beads

Totally, 0.85x AMPure XP Beads were added to the remaining PCR products and mixed for at least 10 times. The PCR tubes were placed at room temperature for 5 min and then placed on the magnetic bead plate until the liquid became clear (about 5 min). The PCR tubes were mixed with 200 μl of 80% alcohol and washed for 30 s, and then, the liquid was carefully sucked out and discarded. The PCR tubes on the magnetic bead plates were dried until the beads were cracked completely (about 5 min). About 17 μl of nuclease-free water was added to the dried beads, and the beads were blended. The PCR tubes were put on the magnetic bead plates for separation, and after the liquid became clear, 15 μl liquid was transferred into the new PCR tube.

### Pyrosequencing Using 16S rDNA Amplicon

Forward primer 515F (5′-GTGCCAGCMGCCGCGGTAA-3′) and reverse primer 806R (5′-GGactachVGGGTWTCTAAT-3′) were used for PCR amplification of V4 region of bacterial 16S rRNA gene. Barcode was synthesized into the sequence using a 7-bp specific sequence. A total of 50 μl PCR reaction system included 25 μl of Phusion High-Fidelity PCR Master Mix with HF Buffer, 3 μl (1 μm) of F/R primers, 10 μl of DNA sample, and 12 μl of ddH_2_O. The PCR system was amplified according to the following conditions: pre-denaturation for 30 s at 98°C, followed by 30 cycles. PCR products were purified with AMPure XP Beads (Beckman Coulter, Indianapolis, IN) and quantified using the Qubit dsDNA HS Assay Kit. After quantification, Illumina NovaSeq 6000 paired-end 2 × 150-bp platform was used for sequencing.

### Quantitative Polymerase Chain Reaction for Histone Deacetylase Type 1

The Gene sequence of HDAC1 was researched in NCBI, and its primer (F: GTGTGGCTCAGACTCCCTATC, R: AGCATCAGCATAGGCAGGTTA) was designed with Primer 5. After the cells were treated, 1 ml TRIzol was added to obtain RNA. The cDNA reverse transcription and QPCR amplification test were performed as follows: thermal denaturation at 95°C for 120 s 1 cycle, degeneration of 95°C for 15 s, anneal extends 60°C for 30 s, and dissolution curve at 60–95°C. Target gene amplification was performed by the computer. The relative expression of the HDAC1 gene was calculated as follows: 2^–△△Ct^ = 2^–[(△Ct) Test–(△Ct) Control]^.

### Sequence Analysis

The data of each sample from the original data according to the barcode sequence and primer sequence were split. After the barcode and primer sequences were cut off, Vsearch v2.4.4 was used to splicing the reads of each sample to obtain the original Tags data (Raw Tags). Simultaneously, the control and filtration of the sequence quality were performed. The criteria for screening low-quality sequences were as follows: the sequences less than 150 bp, the average mass value less than 20, the sequences containing unclear bases, and the mononucleotide repeats containing > 8 bp were screened; the chimeric sequences were also removed and the final valid data (Effective Tags) were obtained.

### Statistics Analysis

Quantitative Insights Into Microbial Ecology (QIIME) software was used to calculate the alpha diversity index of operational taxonomic unit (OTU) level, including Chao1, ACE, PD_whole_tree, Shannon, and Simpson, then, the curve of ranked abundance was formed, and the dilution curve was drawn. The difference analysis of the alpha diversity index between the groups was used to compare OTU abundance and evenness between the samples. The beta diversity analysis was performed using QIMME software to calculate the UniFrac distance measure (Lozupone and Knight, 2005; Lozupone et al., 2007). Principal component analysis (PCA), principal coordinate analysis (PCoA), and non-metric multidimensional scaling (NMDS) maps were drawn for beta diversity analysis of microbial flora structure of different samples.

The *t*-test and the Monte Carlo permutation test were used to draw a box plot to compare UniFrac distance differences between groups. The Kruskal method of the R stats package was used to compare the differences in taxonomic phylum, class, order, family, and genus between samples and groups. In LDA effect size (LEfSe) analysis, the LEfSe default setting was employed to detect the differences in classification units between the groups. In random forest analysis, the R package default setting was used to compare the differences between groups. ANOVA was used to compare tumor volumes and cellular proliferation inhibition rate between groups, and least significant difference (LSD) was served to perform the multiple comparisons. *p-*value < 0.05 was considered to be statistically significant.

## Results

### Berberine and Probiotics Reduced the Growth of HT29 Cells and Colon Cancer in Nude Mice

**FIGURE 1:**
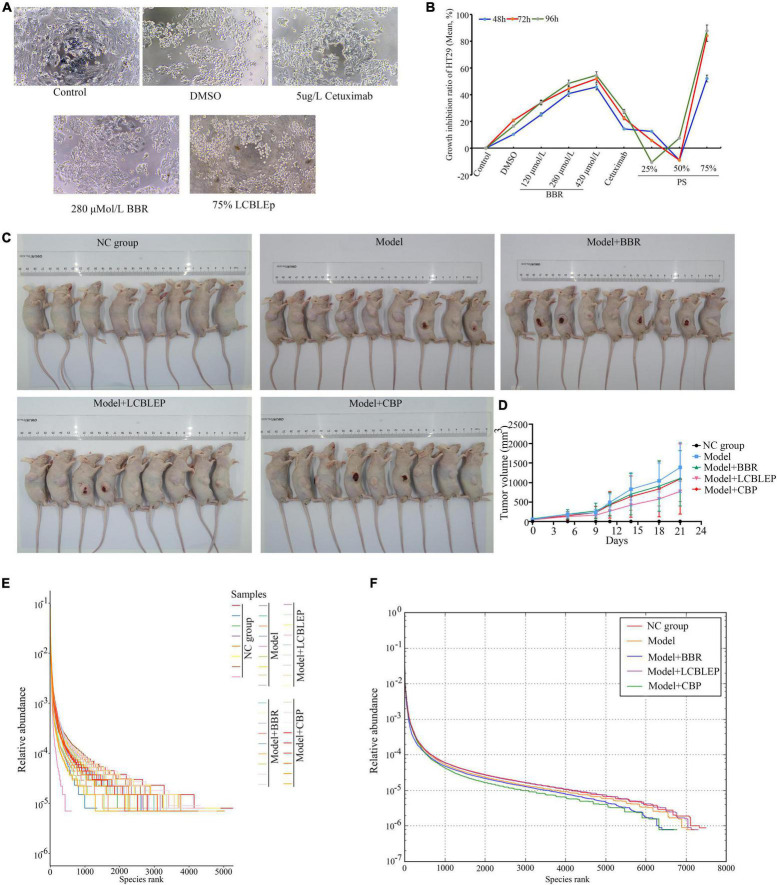
**(A)** Morphological observation of HT29 cells treated with 75% probiotic supernatant (SB) and berberine (200×). **(B)** Growth inhibition ratio of HT29 cells in different conditions through MTT. BBR, berberine; PS, a probiotic (live combined Bifidobacterium, Lactobacillus, and Enterococcus powder) supernatant (diluted with culture medium). **(C)** Establishment of xenograft model of colon cancer in nude mice and treatment of berberine and LCBLEP and CBP. **(D)** Measurement of tumor volume. **(E)** Rank abundance curve of OTU in all samples presenting richness and evenness of community of species. **(F)** Rank abundance curve of OTU in each group presenting richness and evenness of community of species. The sequences were clustered into OTUs with 97% similarity by default. OTU analysis was performed using Vsearch v2.4.4. NC group, normal control (8 nude mice); Model, model of colon cancer (9 nude mice); Model + BBR group, model with berberine (BBR) (9 nude mice); Model + LCBLEP group, model with LCBLEP (9 nude mice); Model + CBP group, model with CBP (9 nude mice) (the same below).

The cultured HT29 cells were treated with BBR or PS with different concentrations, the diverse cellular morphology was observed compared with the control (Figure 1A), and cell growth appeared to be significantly inhibited. We further found that the inhibition of BBR on cell proliferation was concentration-dependent which was not significant in the cells with PS using MTT methods (Figure 1B). The suppression of 75% PS on the proliferation was the most significant. These results revealed that both BBR and probiotics could inhibit the growth of colon cancer cells *in vitro*.

As shown in Figures 1C,D and Table 1, the tumor of the model began to increase in size by 5 days. The treatment of BBR and combination of BBR with probiotics (CBP) reduced the growth of neoplasms to a different extent, especially at 14 days; however, no statistical difference was observed compared with the model. Interestingly, the inhibitory effect of LCBLEP on the tumor growth was obvious, especially at 11—21 days, there was a difference in statistics between LCBLEP and the model group.

**TABLE 1:** The tumor growth volume of each group was compared at different time (*n* = 9).

Days Groups	0 day	5 days	9 days	11 days	14 days	18 days	21 days
Group A (*n* = 8)	0.00 ± 0.00	0.00 ± 0.00	0.00 ± 0.00	0.00 ± 0.00	0.00 ± 0.00	0.00 ± 0.00	0.00 ± 0.00
Group B (*n* = 9)	53.32 ± 14.01	182.00 ± 75.18	226.62 ± 85.33	494.47 ± 219.56	828.44 ± 426.19	1043.50 ± 458.30	1388.74 ± 644.50
Group C (*n* = 9)	68.64 ± 19.13	186.57 ± 120.31	270.69 ± 200.13	440.94 ± 300.95	701.82 ± 532.70	911.07 ± 648.90	1108.13 ± 711.59
Group D (*n* = 9)	45.99 ± 16.90	125.60 ± 49.16	163.40 ± 54.12	266.65 ± 107.86*	420.71 ± 131.57*	579.12 ± 180.78*	773.03 ± 261.63 *
Group E (*n* = 9)	51.54 ± 23.46	149.65 ± 100.75	231.37 ± 155.80	426.63 ± 339.11	639.99 ± 550.40	835.55 ± 716.93	1093.91 ± 903.57

Group A, normal control; Group B, model of colon cancer; Group C, model with berberine (BBR); Group D, model with probiotics preparation (LCBLEP); Group E, model with CBP. *P<0.05 vs. Group B.

### Berberine and Probiotics Affected Richness and Evenness in the Gut Microbiota

As observed in Figures 1E,F, the growth of colon cancer cells had an influence on the abundance of intestinal microbiota. Compared with the normal control, the richness and evenness of gut microbiota in the model nude mice were decreased, which was affected by the treatment of BBR and LCBLEP. The supplement of probiotics (LCBLEP) significantly increased both the richness and evenness, whereas the effects of alone use of the BBR and the CBP on the enhancement of richness and evenness were not obvious.

### Berberine and Probiotics Significantly Improved the Composition of Gut Microbiota in Nude Mice

**FIGURE 2:**
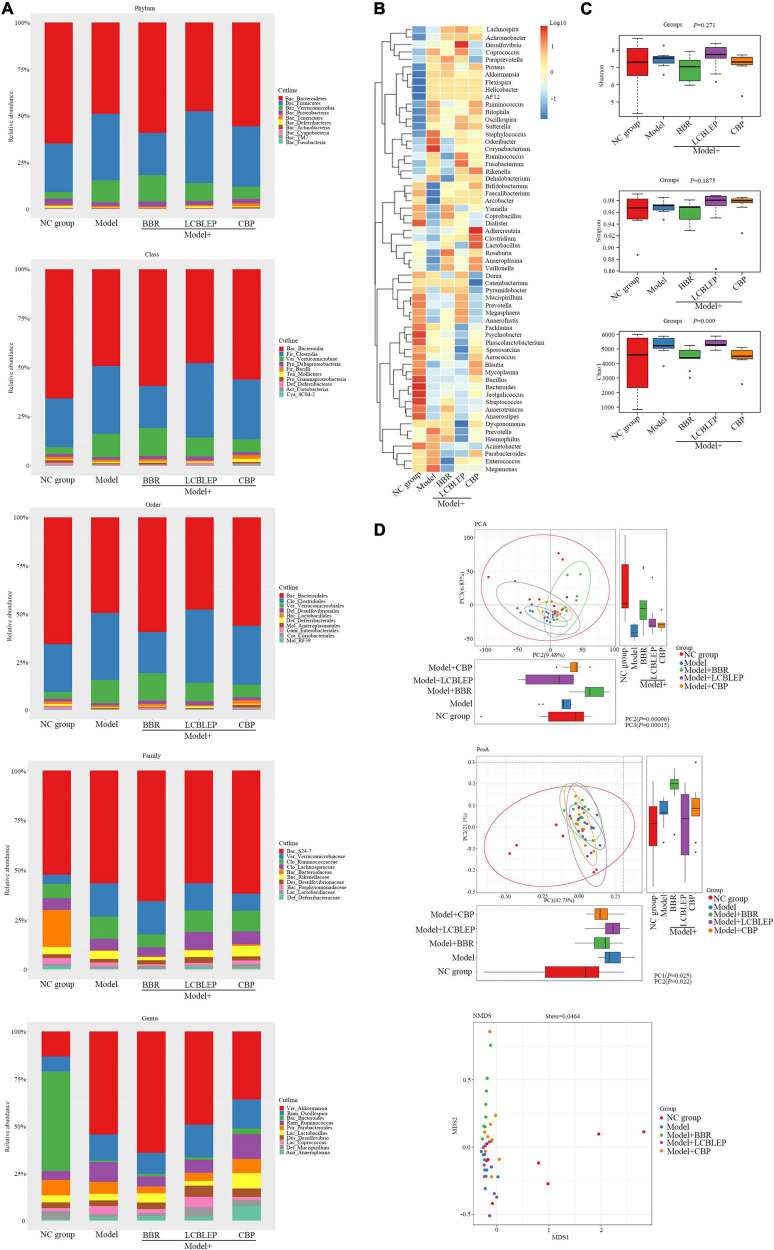
**(A)** Histograms of relative abundance of species composition at phylum, class, order, family, and genus level, respectively. **(B)** Clustering heat maps based on genus level were performed to reveal the similarity between samples and the similarity of community composition at the genus level. **(C)** Box diagram of alpha diversity was analyzed through assessing the species richness and community diversity of the microbiome by Shannon, Simpson, and Chao1. **(D)** The beta diversity was used to compare the differences between multiple groups of samples by the multivariate statistical method including principal component analysis (PCA), principal coordinate analysis (PCoA), and non-metric multidimensional scaling (NMDS). The horizontal and vertical box diagrams were the distribution of the values of the different groups on the first and the second principal coordinates. Each dot represented a sample, the same color was from the same group, and distance reflected sample similarity.

As shown in Figure 2A, the gut microbiota was analyzed according to the classification of phylum, class, order, family, and genus. At the level of phylum, the relative abundance of *Firmicutes*, *Verrucomicrobia*, and *Clostridia* was significantly increased in the xenograft model of colon cancer in nude mice. On the contrary, the abundance of *Bacteroidete*s and *Proteobacteria* was decreased in the model, which was improved by the treatment of BBR instead of LCBLEP. The addition of probiotics reduced the relative abundance of *Verrucomicrobia*. The CBP also promoted the relative level of *Bacteroidete*s but reduced the level of *Verrucomicrobia*, which was consistent with that of probiotics. In class, compared with the control, the relative abundance of *Bacteroides* was significantly reduced in the model, which was reversed by the treatment of BBR. The upregulated abundance of *Clostridia* and *Verrucomicrobiae* was observed in the model; however, the reduction of the level of *Clostridia* instead of *Verrucomicrobiae* existed in the treatment of BBR. The ingestion of probiotics decreased the relative level of *Verrucomicrobiae*. Interestingly, the CBP increased the relative abundance of *Bacteroidia* and decreased the level of both *Clostridia* and *Verrucomicrobiae* compared with the model. These phenomena in the class were consistent with that in the classification of the order. In the family, promotion of relative abundance of *Verrucomicrobiaceae* and *Ruminococcaceae* and downregulation of relative level of *Bacteroidaceae* were found in the model in contrast to the control. The level of *Ruminococcaceae* was reduced by BBR. The treatment of LCBLEP only reduced the relative level of *Verrucomicrobiaceae*, which was consistent with that of the CBP. In genus, the relative abundance of *Akkermansia*, *Oscillospira*, and *Ruminococcus* was significantly increased in the model, whereas the level of *Bacteroides* was dramatically decreased compared to the control. The CBP significantly decreased the level of *Akkermansia*, followed by the LCBLEP. The CBP also obviously increased the level of *Bacteroides*, followed by the BBR and LCBLEP, but BBR reduced the relative abundance of *Ruminococcus*, followed by the LCBLEP.

Next, we performed a standardized cluster analysis to understand the similarity between samples and the similarity of gut microbiota composition at the genus level, and the results are shown in Figure 2B. The composition of the gut microbiota was divided into two categories in general. Compared with the control, *Staphylococcus*, *Odoribacter*, and *Corynebacterium* had a very high abundance and played a similar role as a class, whereas *Prevotella*, *Megamonas* and *Haemophilus*, *Acinetobacter*, *Parabacteroides*, and *Enterococcus* played the other similar role as an alternative class. Interestingly, these microbes with high abundance were downregulated by BBR and LCBLEP. This effect was observed in the microbes with low abundance which were also mediated by BBR or LCBLEP, especially the CBP. Some microbes that include *Adlercreutzia*, *Clostridium*, and *Lactobacillus* were promoted by the treatment of CBP.

### Dysregulated Diversity of Intestinal Microbiota Was Modulated by Berberine and Probiotics

Alpha diversity, an analysis of species diversity in a single sample, is calculated and assessed by the richness and diversity of the microbe through a series of statistical indices. The diversity index measures the heterogeneity of a community. So far, commonly used measure indictors for alpha diversity include Chao1, Shannon, and Simpson. Chao1 is used to measure community richness, whereas Shannon and Simpson are adopted to assess community diversity.

Wilcoxon test was conducted for each index of alpha diversity, and then, the alpha diversity index with significant differences was screened. The results are revealed in Figure 2C, although no statistical difference in diversity between groups was found by Shannon and Simpson, the Chao1 showed that the alpha diversity between groups was significantly different in statistics (*p* < 0.01).

Inconsistent with alpha diversity, beta diversity aimed to compare the differences between multiple groups of samples. Principal component analysis (PCA) and principal coordinate analysis (PCoA) were used to analyze the difference in beta diversity between groups as the two kinds of multivariate statistical methods. As shown in Figure 2D, the differences in beta diversity between groups were significant in statistics by the analysis of PCA and PCoA (*p* < 0.05). Compared with the control, the beta diversity in the model group was significantly reduced, which was improved by the treatment of BBR and probiotics.

### Berberine and Probiotics Significantly Affected the Biomarker of the Gut Microbe in Nude Mice With Colon Cancer

**FIGURE 3:**
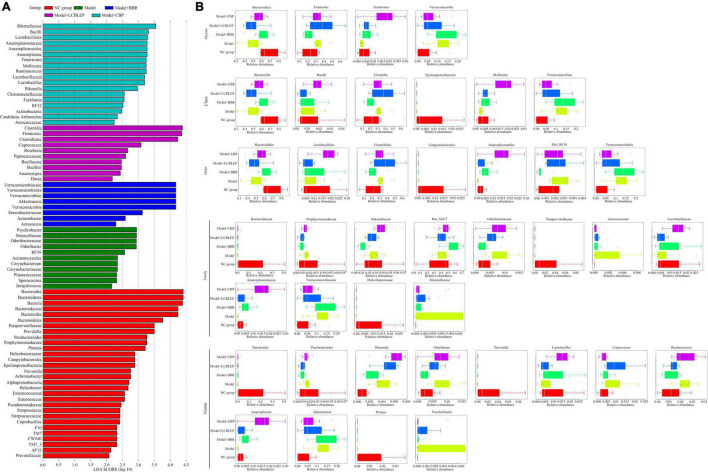
**(A)** Species biomarkers were selected for comparison between groups through LEfSe analysis. **(B)** Box diagram of species with significant differences between groups at phylum, class, order, family, and genus level. The different colors in the figure represented the species with significant differences between groups.

Biomarkers, namely, the dominant species with significant difference between groups, were screened through LEfSe analysis. As shown in Figure 3A, the biomarkers in the model group, included *Psychrobacter*, *Moraxellaceae*, *Odoribacteraceae*, and *Odoribacter*. *Verrucomicrobiaceae*, *Verrucomicrobiales*, *Verrucomicrobiae*, *Verrucomicrobia*, and *Akkermansia*, were the primary biomarkers in the group treated with BBR. On the contrary, *Clostridia*, *Firmicutes*, *Clostridiales*, and *Coprococcus* were the markers in the mice intervened with LCBLEP. However, the biomarkers in the group treated with CBP were significantly different from the other groups, including *Rikenellaceae*, *Bacilli*, *Lactobacillales*, *Anaeroplasmataceae*, *Anaeroplasmatales*, *Anaeroplasma*, *Tenericutes*, *Mollicutes*, *Ruminococcus*, *Lactobacillaceae*, *Lactobacillus*, and *Rikenella*.

Next, the biomarkers were selected between groups according to the species classification level (Figure 3B). In phylum, the dominant biomarkers included *Bacteroidetes*, *Firmicutes*, *Tenericutes*, and *Verrucomicrobia*. These species were dysregulated in the nude mouse model of colon cancer but modulated by BBR or LCBLEP. BBR significantly promoted the relative abundance of *Bacteroidetes* but inhibited the level of *Firmicutes*, which was consistent with that of CBP. In class, the relative abundance of *Bacteroidia* and *Mollicutes* was increased by BBR, with decreased *Clostridia*. The treatment of CBP obviously promoted the relative level of *Mollicutes* but decreased the abundance of *Verrucomicrobiae*. Similarly, these dominant species in the order were *Bacteroidales*, *Lactobacillales*, *Clostridiales*, *Campylobacterales*, *Anaeroplasmatales*, *Mol_RF39*, and *Verrucomicrobiales*. The abundance of *Anaeroplasmatales* was dramatically increased by the CBP, with decreased *Verrucomicrobiales.* In family, these biomarkers reduced by BBR included *Porphyromonadaceae*, *Rikenellaceae*, and *Odoribacteraceae*, as well as *Lactobacillaceae* and *Anaeroplasmatacea*e were increased. Compared with the model, reduction of *Porphyromonadaceae*, *Odoribacteraceae*, *Verrucomicrobiaceae*, and *Moraxellaceae* by probiotics was found, with increased *Anaeroplasmataceae*. Surprisingly, no influence of BBR or LCBLEP on the markers including *Bacteroidaceae*, *Paraprevotellaceae*, and *Helicobacteraceae* was observed in contrast to the model group. Similarly, the effect of BBR or LCBLEP on the biomarkers in genus involving *Bacteroides*, *Prevotella*, and *Proteus* was also not observed compared with the model. BBR decreased the abundance of *Parabacteroide*s, *Rikenella*, *Odoribacter*, *Coprococcu*s, *Ruminococcus*, and *Psychrobacter*, with increased *Anaeroplasm*a and *Lactobacillus*, whereas the relative level of *Parabacteroides*, *Odoribacter*, *Ruminococcus*, *Akkermansia*, and *Psychrobacter* was reduced by treatment of LCBLEP compared to the model group. Besides, we found that the relative abundance of *Moraxellaceae* and *Psychrobacter* was dramatically high and dominant biomarkers in the gut of colon cancer.

**FIGURE 4:**
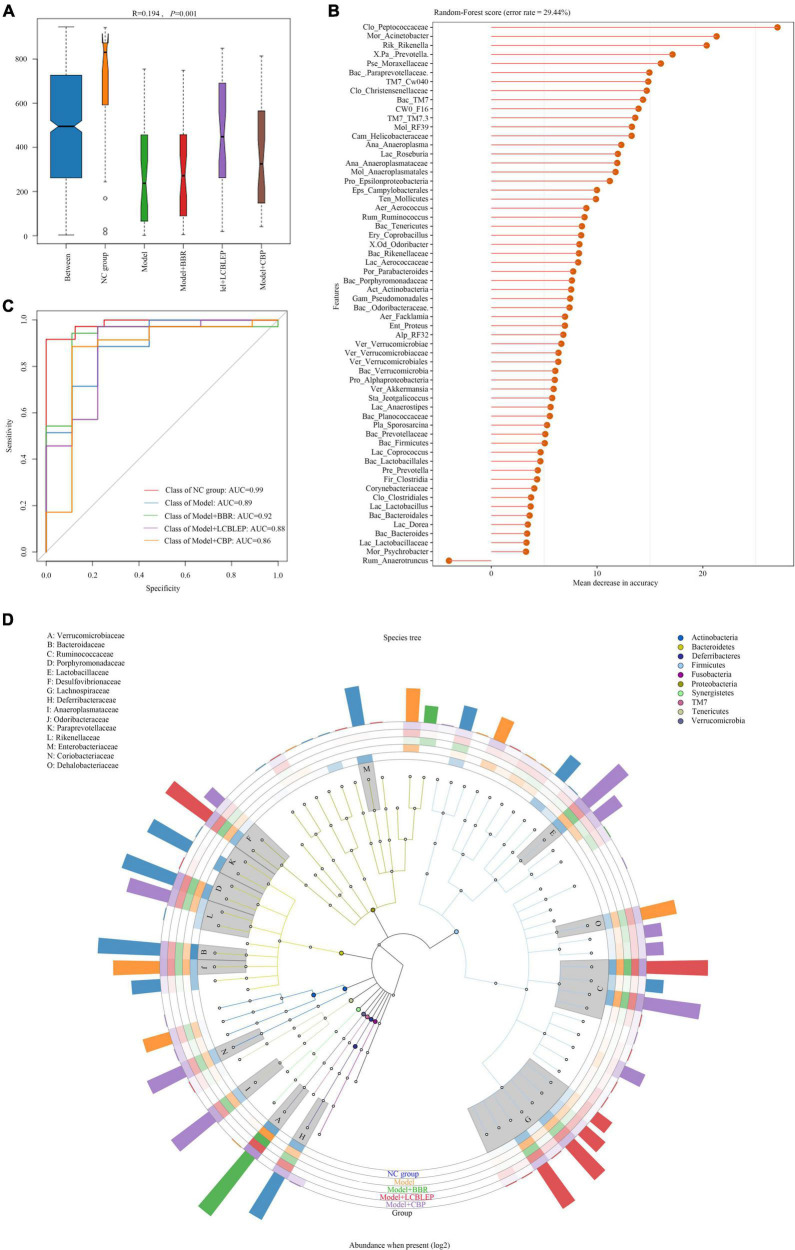
**(A)** Species biomarkers were tested through analysis of similarities (ANOSIM) which was used to detect whether the difference between groups was significantly greater than the difference within groups, so as to judge whether the grouping was meaningful. R-value was used to compare whether there are differences between groups. If R-value was between (−1, 1) and greater than 0, the difference between groups was greater than that within groups. R-value < 0 indicated the inter-group difference was smaller than the intra-group difference. **(B)** Random forest classification tree with high accuracy was performed to effectively classify and predict the grouped samples. **(C)** The abscissa was the level of importance, and the ordinate was the species name in the order of importance. Receiver operating characteristic (ROC) curves were drawn at genus level. **(D)** GraPhlan figure, a map of sample communities in the evolutionary tree of species, was used to easily observe the dominant species.

Whether the grouping of these markers was meaningful? Analysis of similarities (ANOSIM) was used to test whether the difference between groups was significantly greater than the difference within groups. As shown in Figure 4A, the difference between groups was higher than that within groups (*R* = 0.194, *p* < 0.05). However, what were the predominant species used for classification. A random forest classification tree was adopted. As revealed in Figure 4B, *Peptococcaceae* was the dominant microbe playing important role in the grouping, followed by *Acinetobacter* and *Rikenella*. How effective were these major biomarkers in a grouping? We found that these biomarkers demonstrated some accuracy in the diagnosis of all groups (all AUC > 0.85) (Figure 4C).

In this study, we performed an evolutionary tree of species by GraPhlan to find the dominant species more visually in each group (Figure 4D). *Odsoribacteraceae*, *Dehalobacteriaceae*, and *Proteobacteria* were the predominant intestinal microbes in colon cancer, whereas only *Verrucomicrobiacea*e was dominant in the gut treated by BBR. The main species in the gut treated by LCBLEP were *Desulfovibrionaceae*, *Ruminococcaceae*, and *Lachnospiraceae*, which was partly shared with that of the intestine intervened by CBP where *Anaeroplasmataceae*, *Coriobacteriaceae*, and *Rikenellaceae* were also predominant.

### Berberine and Probiotics Modulated the Metabolic Function of Intestinal Microbe in Nude Mice With Colon Cancer

**FIGURE 5:**
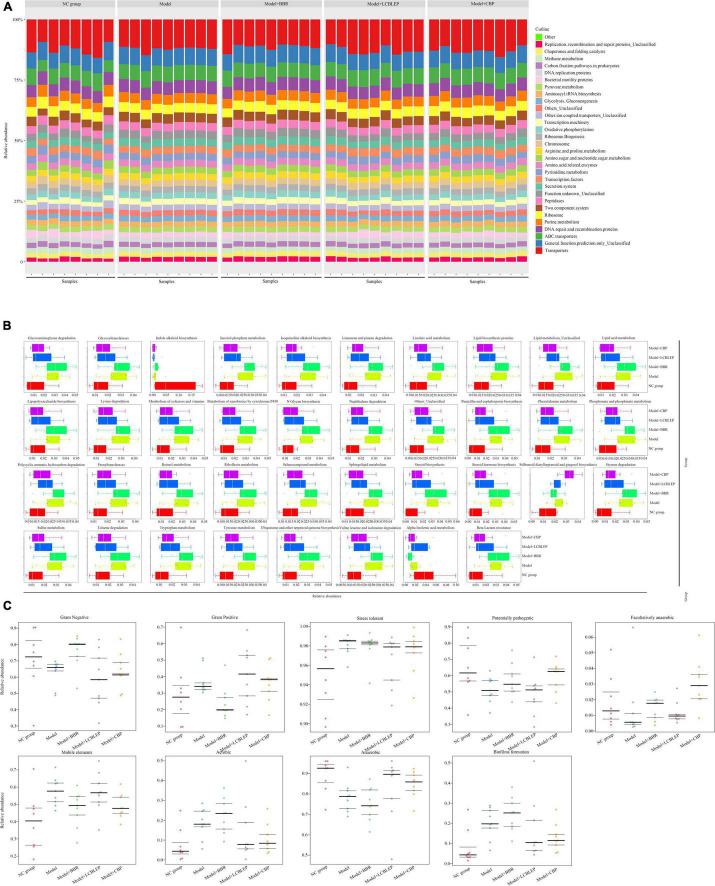
**(A)** Prediction of microbial metabolic function was performed through PICRUSt based on 16S rDNA sequence, and the enrichment of functional genes in KEGG Pathways with levels 3 was obtained, with a histogram of the top 30 metabolic pathways with the highest abundance. **(B)** Box diagram for function prediction of KEGG level3 of differences between groups in genus level. **(C)** Comparison of phenotypic classification for microbial metabolic function based on BugBase, and the three lines represented the upper, the mean, and the lower quartiles from top to bottom.

Intestinal microbe plays an important role in regulating intestinal cell metabolism. We performed a PICRUSt function prediction based on a 16S rDNA sequence to obtain enrichment of functional genes of KEGG metabolic pathways in level 3. The results are presented in Figure 5. The top 30 metabolic pathways with the highest abundance are shown in Figure 5A, and replication, recombination, and repair were the crucial genes in the Kyoto Encyclopedia of Genes and Genomes (KEGG) pathway predicted by PICRUSt, followed by transporters, DNA repair, and recombination.

Next, we established box diagrams of significant differences for function prediction with KEGG level 3 of species between groups at the genus level. As revealed in Figure 5B, compared with the control, the metabolisms in the gut of nude mice implanted with colon cancer cells were aberrantly active except for indole alkaloid biosynthesis and alpha-linolenic acid metabolism (*p* < 0.05), which was significantly repressed by the intervention of the LCBLEP. However, the treatment of BBR instead of LCBLEP inhibited the biosynthesis of stilbenoid diarylheptanoid and gingerol. Interestingly, the influence of LCBLEP on the metabolisms of gut microbe was similar to that of CBP.

Another analysis of the metabolic function of gut microbiota was performed through the comparison of phenotypic classification based on BugBase (an online 16S function prediction tool), and the results are shown in Figure 5C. These phenotypes included Gram-positive, Gram-negative, biofilm formation, pathogenicity, mobile elements, oxygen requirements, and oxidative stress tolerance, involving anaerobic bacteria, aerobic bacteria, and facultative bacteria. We found that Gram-negative bacteria, potentially pathogenic bacteria, facultative bacteria, and anaerobic bacteria were decreased in the gut of nude mice with colon cancer compared to the control (*p* < 0.05), whereas Gram-positive bacteria, stress tolerance, mobile elements, aerobic bacteria, and biofilm formation were upregulated (*p* < 0.05). On the contrary, BBR upregulated the relative abundance of Gram-negative bacteria, potential pathogenic bacteria, facultative bacteria, aerobic bacteria, and biofilm formation and downregulated the level of Gram-positive bacteria, mobile elements, and anaerobic bacteria. These effects of BBR were opposite to that of LCBLEP. However, the influence of CBP on the phenotype of intestine microbe was different from that of BBR or LCBLEP. CBP partly neutralized the effect of BBR and LCBLEP, such as gram-negative and -positive bacteria, aerobic bacteria, anaerobic bacteria, and biofilm formation. Interestingly, significant upregulation of facultative bacteria and downregulation of mobile elements were found in the gut treated with CBP, compared to that of BBR or LCBLEP. Besides, BBR, LCBLEP, and CBP inhibited the relative level of stress tolerance, especially LCBLEP and CBP.

### Berberine Inhibited the Growth of Colon Cancer Through Regulation of Metabolic Product Sodium Butyrate Mediating Histone Deacetylase Type 1 Expression

**FIGURE 6:**
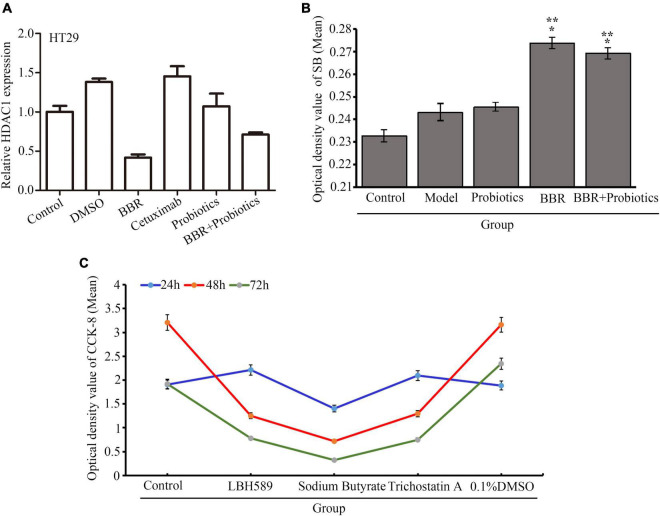
**(A)** Relative level of HDAC1 mRNA in the HT29 cells with different conditions. **(B)** Expression of sodium butyrate (SB) in the serum of mice treated with BBR and probiotics (LCBLEP). **(C)** Description of proliferation in the HT29 cells treated with three kinds of HDAC inhibitors at different times using CCK-8. *p < 0.05 vs. control, **p < 0.05 vs. the model group.

In the process of colon cancer, although amino acids and other bacterial metabolites increased, the production of butyrate was decreased. In fact, many beneficial species that maintain intestinal homeostasis by producing butyrate, including Bifidobacterium, Roseburia, and Faecalibacterium prausnitzii, were reduced in patients with CRC (Fukuda et al., 2011). Butyrate, a regulator of epigenetic modifications, is responsible for downregulating the acetylation of histones and is considered a carcinostatic agent. We observed that level of histone deacetylase type 1 (HDAC1) mRNA was reduced in the HT29 cells treated with BBR or PS compared with the control (*p* < 0.05), whereas this phenomenon was not found in the cells with cetuximab treatment (Figure 6A). Interestingly, the decreased level of HDAC1 mRNA in the BBR treatment group was lower than that of PS or BBR combined with PS (*p* < 0.05), which brought us a clue that BBR might upregulate or increased some products leading to suppression of HDACs such as HDAC1. As shown in the serum of mice (Figure 6B), the mice treated with BBR revealed a significantly increased concentration of sodium butyrate (SB) in serum compared with the model (*p* < 0.05) followed by the combination of BBR with LCBLEP. Next, we selected three kinds of HDAC inhibitors, LBH589, sodium butyrate, and trichostatin A. Our observation found that the inhibitory effect of SB on the proliferation of HT29 cells was stronger than LBH and TSA, and this effect was time-dependent (Figure 6C).

## Discussion

Colorectal cancer is one of the most common types of cancer and the third leading cause of cancer-related death (Fitzmaurice, 2018). The human colon is a complex microbial ecosystem. Intestinal epithelial cells with high regeneration pressure are frequently in contact with nutrients and microbiota and are prone to malignant transformation. Extensive studies have revealed the key role of the microbiome in colon tumorigenesis (Roberti et al., 2020; Yang et al., 2022). Dysbiosis of gut microbe has been demonstrated to be beneficial to the risk of tumor formation (Garrett, 2019; Molska and Reguła, 2019; Nejman et al., 2020; Pothuraju et al., 2021). In 1975, researchers first discovered the association between gut flora and CRC (Weisburger et al., 1975). Antibiotics in CRC transplanted mice not only resulted in decreased *clostridium* but also repressed cancer cell proliferation and overall tumor growth (Yamaoka et al., 2018; Oh et al., 2019). Compared with healthy people, patients with CRC had less diversity and significantly decreased abundance of beneficial bacteria in their gut. These dysregulated microbes can activate chronic inflammation releasing various cytokines and producing a lot of exotoxins and endotoxins directly or indirectly inducing DNA damage, genomic instability, tumorigenesis, and adenocarcinoma invasion (Irrazabal et al., 2020; Loke et al., 2020; Abu-Ghazaleh et al., 2021). In our study, we found that obvious dysbiosis has existed in the gut of nude mice with colon cancer cells. Therefore, these pieces of evidence have suggested that regulation of intestine microbe is an important strategy to prevent and cure the occurrence and development of colon cancer.

Increasingly extensive clinical and experimental data suggest that some Chinese herbal extracts play a therapeutic role by regulating the abundance of intestinal microbiota. BBR, an alkaloid extracted from Rhizoma Coptidis, is an anti-inflammatory drug used to treat infections in the digestive tract. Recent researches have demonstrated its potent antitumor activity (Liu et al., 2020; Song D. et al., 2020; Zhang Q. et al., 2020). Interestingly, BBR has been found to regulate the intestinal microbe of rats fed with a high-fat diet, thus improving their metabolic status (Sun et al., 2016), which is similar to that of probiotics. Probiotics, now widely regarded as biological therapies, have a variety of biological benefits to host health, including anti-bacterial activity, regulating the immune system, inhibiting colitis, and preventing CRC. In patients with CRC, direct supplementation of probiotics can promote the effect of CRC-related therapies through the regulation of intestinal microbiome (Molska and Reguła, 2019; Fong et al., 2020; Torres-Maravilla et al., 2021). This suggests that mediation of dysbacteriosis may be one of the new antitumor mechanisms of BBR (Habtemariam, 2020). However, it is not known whether the combination of BBR and probiotics has a synergistic effect on the inhibition of colon cancer growth.

Live combined *Bifidobacterium*, LCBLEP, is an oral probiotic preparation widely used in a clinic. Consistent with the previous study, our observation has found that, although there is no difference in statistics for BBR, both BBR and probiotics can inhibit the growth of colon cancer cells to some extent, suggesting that the suppressive effect of BBR is lower than that of probiotics. Further study has found that, although the treatment of the BBR on the enhancement of richness and evenness was not obvious in contrast to that of the LCBLEP, composition of microbe at each classification level can be modulated by BBR in which the abundance of *Bacteroidetes* and *Proteobacteria* is significantly increased. In our study, we found dramatically decreased *bacteroides* in the gut of colon cancer mice, which was increased by BBR and LCBLEP, especially CBP. These suggest that *Bacteroides* is a beneficial bacterium that suppresses the growth of colon cancer. Some studies have demonstrated that *Bacteroidetes* can regulate the production of E-cadherin, nuclear factor-κB, and STAT3, and its abundance is decreased in CRC (Hwang et al., 2020; Jiang et al., 2020; Zhang W. et al., 2020).

In addition, we observed that BBR significantly increased the abundance of *Roseburia* which was dramatically reduced in CRC (Fukuda et al., 2011), suggesting that *Roseburia* is one of the beneficial species that maintain intestinal flora homeostasis by producing butyrate, and this efficacy is further enhanced by the CBP. It means that the improvement of intestinal dysbiosis by a combination of BBR with probiotics is stronger than that by BBR and probiotics alone. Just because of this, both BBR and probiotics can improve the alpha biodiversity and beta biodiversity in the gut with colon cancer cells. Once the gut microbiota remains in dysbiosis, the diversity of beneficial symbiotic bacteria is reduced, resulting in the production of various bacteriotoxins or increased exposure of colon epithelial cells to carcinogens (Meng et al., 2018). Recent research also reveals that BBR can reverse the structural and numerical changes in the intestine microbiota under pathological conditions (Habtemariam, 2020). Consistent with our study, BBR and LCBLEP can alter the biomarkers in the gut, especially the CBP, suggesting that the combined BBR and probiotics had a greater regulatory effect on the bacterial community disorder than BBR and probiotics alone.

Many studies have shown that obesity is considered a risk factor for CRC (Chen et al., 2021), and recent research suggests that an increased intestinal *Firmicutes*/*Bacteroides* ratio is the hallmark of obesity (Kim et al., 2021). We speculated that regulation of the *Firmicutes*/*Bacteroides* ratio may be a strategy to control the growth of colon cancer. Our results demonstrated that the increased abundance of *Firmicutes* and decreased *Bacteroides* existed in the model planted with colon cancer, which was reversed by BBR and CBP. Besides, some pro-inflammatory bacteria such as *Ruminococcus*, *Peptococcaceae*, *Lactobacillus*, and anti-inflammatory *Bifidobacterium* exist within the gut (Kim et al., 2021). Decreased *Ruminococcus* and increased *Lactobacillus* were observed in the mice treated with BBR, suggesting that BBR can regulate the proportion of inflammatory bacteria. *Clostridiales*, *Bifidobacterium*, and *Lachnospiraceae* are beneficial bacterial taxa (Zhou et al., 2021), and both LCBLEP and CBP promoted the relative abundance of *Clostridiales* and *Lachnospiraceae.* These data suggest that BBR and LCBLEP mediate the dysbacteriosis in the gut of nude mice with colon cancer, but the effect of the combination of BBR with probiotics may be more potent.

In addition, we found other bacteria increased by BBR, such as *Roseburia*, and thus, we considered that *Roseburia* is also useful in suppression of colon cancer. An increasing number of evidence reveals that anti-cancer short-chain fatty acids (SCFAs) were produced by beneficial bacteria including *Eubacterium*, *Roseburia*, and *Rikenella* (Shi et al., 2017; Zheng et al., 2020), and these bacteria were reduced in patients with CRC (Wang et al., 2012). Similarly, reduced *Lachnospira* was observed in the gut of colon mice or patients with cancer (Clos-Garcia et al., 2020), which was increased by BBR and LCBLEP but not CBP. However, CBP can dramatically increase the abundance of *Adlercreutzia*, *Lactobacillus*, and *Rikenella* families, compared with that of BBR and LCBLEP. These results have concluded that the combination of BBR and probiotics has a greater effect on the regulation of bacterial dysregulation in the process of inhibiting colon cancer growth.

Healthy gut microbiota is described by metabolic function. Antimicrobial peptides and immunomodulatory compounds produced by the beneficial microbiome can affect the mucosal immune system, regulating their anticancer effects. Microbiota and its related metabolites are not only closely related to carcinogenesis by inducing inflammation and immune disorders, leading to genetic instability (Kompella and Vasquez, 2019; Tan et al., 2021). Therefore, the metabolic pathways with the highest abundance including replication, recombination, and DNA repair were observed in our study. These active metabolic functions were found in the gut of colon cancer mice, including glycosaminoglycan degradation, glycosyltransferases, lipopolysaccharide biosynthesis, lipid biosynthesis proteins, N glycan biosynthesis, etc., and these were reversed by LCBLEP or CBP instead of BBR which only downregulated the stilbenoid diarylheptanoid and gingerol biosynthesis. However, indole alkaloid biosynthesis and alpha-linolenic acid metabolism were reduced in colon cancer and mediated by both BBR, LCBLEP, or CBP. These data suggest that the effects of probiotics on the metabolic function of microbe in the colon cancer mice are higher than that of the BBR.

However, the phenotypic effects of BBR on the metabolic functions of bacterial community are very significant. Our observations reveal that BBR can obviously promote the abundance of microbiota including G^+^ bacteria, facultatively anaerobic bacteria, aerobic bacteria and repress the G^–^ bacteria, mobile elements, and anaerobic bacteria, compared with the model. *Bacteroidetes* are G- bacteria and *Firmicutes* are G +, combining with the effect of BBR on the other bacteria including *Verrucomicrobiaceae*, *Akkermansia*, and *Enterobacteriaceae*, etc. These indicate that BBR has a broad spectrum of regulation on bacterial species.

Biofilm formation can destroy the mucus layer of the colon, strengthening cytotoxicity or genotoxicity through enhancement of colonic epithelial invasion, inflammation, and abnormal immune response (Dejea and Sears, 2016). These phenomena eventually result in malignant proliferation and colorectal cancer. In our study, although BBR increased the abundance of biofilm formation, LCBLEP and CBP significantly decreased the level, suggesting that the combination of BBR with probiotics has an effect on the metabolic function of gut microbiome in nude mice with colon cancer.

An increasing number of extensive evidence has demonstrated that metabolites of gut microbes play vital roles in maintaining healthy intestinal homeostasis and preventing colon carcinogenesis. Butyrate can serve as a fuel source for intestinal epithelial cells (Donohoe et al., 2011) and have anti-inflammatory effects. Bifidobacterium, Roseburia, and Faecalibacterium prausnitzii are beneficial species for maintaining the homeostasis of the intestinal microbe by producing butyrate, which can be mediated by BBR. These suggest that sodium butyrate upregulated by BBR changes the patterns of histone modification of some genes participating in the inhibition of cancer growth. However, less research has been done on the relationship between the microbiome and epigenetic changes in CRC. Some study has found that butyrate induced the expression of cell cycle regulation genes (CCND3 and CDKN1A) in intestinal cells. Infection with *L. monocytogenes* causes H3K18 deacetylation of many genomic proteins in colon cells, including SMAD1, IRF2, SMARCA2, and CXCL12 (Sabit et al., 2019). Reduced butyrate can disrupt intestinal barrier function, causing immune dysregulation and leading to cell proliferation, which leads to the development of CRC. In this case, reducing the abundance of *Clostridium* in the intestinal microbe and increasing butyric-producing bacteria may be a treatment strategy for CRC. Furthermore, our results have also found that probiotic was inferior to BBR in increasing butyrate production, suggesting that BBR inhibits the growth of colon cancer cells through the regulation of SB production and HDAC1 expression.

In conclusion, BBR and probiotics can reduce the growth of colon cancer cells, with more potent effect of the latter. Besides, BBR and probiotics can mediate the composition, structure, abundance, biological diversity, and metabolic function of gut microbiome in nude mice with colon cancer, which is more significant in the mice treated with the combination of BBR with probiotics. Therefore, BBR can also be used as a regulator of intestinal microbiome similar to the probiotics. However, the influence of combination of the two on the growth of neoplasm in nude mice is not obvious, suggesting that the combination of BBR and probiotics has no advantage in inhibiting tumor growth compared with drug alone. Besides, BBR instead of probiotics can significantly increase the level of SB production inhibiting the HDAC1 expression, and the inhibitory effect of SB on the growth of colon cancer cells was stronger than LBH and TSA. Based on the current research results, we believe that the mechanism of BBR and probiotics inhibiting the growth of colon cancer cells should be inconsistent. BBR inhibits the growth either through its direct cytotoxic effects or by increasing SB production, while the mechanisms by which probiotics inhibit the growth of cancer cells are likely to work through something other than increased SB production, such as production of SCFAs (Zheng et al., 2020), regulation of macrophages, or relative signaling pathways (Fan et al., 2021), etc.

Unfortunately, no drug positive control for *in vivo* experiments was performed in our design. In addition, the comparison between LCBLEP and other control probiotic strains with proven anti-colon cancer effects, and the performance of cytotoxicity assay of probiotics on normal cells and other different cancer cell lines are our negligence in this study, which will be considered in further research.

## Data Availability Statement

The authors acknowledge that the data presented in this study must be deposited and made publicly available in an acceptable repository, prior to publication. Frontiers cannot accept a manuscript that does not adhere to our open data policies.

## Ethics Statement

The animal study was reviewed and approved by the Laboratory Animal Ethics Committee, Shenzhen University Health Science Center.

## Author Contributions

CH was responsible for the research protocol design and collection of data. YS was responsible for the verification of manuscripts. S-RL, Z-XC, and H-FL were responsible for figure making and layout. W-ZS was responsible for the procurement of reagents or materials. All authors contributed to the article and approved the submitted version.

## Conflict of Interest

The authors declare that the research was conducted in the absence of any commercial or financial relationships that could be construed as a potential conflict of interest.

## Publisher’s Note

All claims expressed in this article are solely those of the authors and do not necessarily represent those of their affiliated organizations, or those of the publisher, the editors and the reviewers. Any product that may be evaluated in this article, or claim that may be made by its manufacturer, is not guaranteed or endorsed by the publisher.
